# Penetrance of eye defects in mice heterozygous for mutation of *Gli3 *is enhanced by heterozygous mutation of *Pax6*

**DOI:** 10.1186/1471-213X-6-46

**Published:** 2006-10-09

**Authors:** Paulette A Zaki, J Martin Collinson, Junko Toraiwa, T Ian Simpson, David J Price, Jane C Quinn

**Affiliations:** 1 Genes and Development Group, University of Edinburgh, Hugh Robson Building, George Square, Edinburgh, EH8 9XD, UK; 2 School of Medical Sciences, Institute of Medical Sciences, University of Aberdeen, Aberdeen AB25 2ZD, UK

## Abstract

**Background:**

Knowledge of the consequences of heterozygous mutations of developmentally important genes is important for understanding human genetic disorders. The *Gli3 *gene encodes a zinc finger transcription factor and homozygous loss-of-function mutations of *Gli3 *are lethal. Humans heterozygous for mutations in this gene suffer Greig cephalopolysyndactyly or Pallister-Hall syndromes, in which limb defects are prominent, and mice heterozygous for similar mutations have extra digits. Here we examined whether eye development, which is abnormal in mice lacking functional Gli3, is defective in *Gli3*^+/- ^mice.

**Results:**

We showed that *Gli3 *is expressed in the developing eye but that *Gli3*^+/- ^mice have only very subtle eye defects. We then generated mice compound heterozygous for mutations in both *Gli3 *and *Pax6*, which encodes another developmentally important transcription factor known to be crucial for eye development. *Pax6*^+/-^; *Gli3*^+/- ^eyes were compared to the eyes of wild-type, *Pax6*^+/- ^or *Gli3*^+/- ^siblings. They exhibited a range of abnormalities of the retina, iris, lens and cornea that was more extensive than in single *Gli3*^+/- ^or *Pax6*^+/- ^mutants or than would be predicted by addition of their phenotypes.

**Conclusion:**

These findings indicate that heterozygous mutations of *Gli3 *can impact on eye development. The importance of a normal *Gli3 *gene dosage becomes greater in the absence of a normal *Pax6 *gene dosage, suggesting that the two genes co-operate during eye morphogenesis.

## Background

The zinc-finger transcription factor Gli3 is required for normal limb, brain and eye development. In humans, a number of different mutations to the *GLI3 *allele can cause Greig cephalopolysyndactyly (GCPS) or Pallister-Hall Syndrome (PHS) [1,2]. Clinical features of GCPS include polydactyly, syndactyly, ocular hypertelorism and macrocephaly; those of PHS include polydactyly, imperforate anus, renal abnormalities, hypothalamic hamartoma and pituitary dysplasia. Mutations of the *Gli3 *gene in mice cause the *extra-toes *(*Xt*) phenotype [3]. Johnson [4] first reported a comprehensive analysis of the developmental anatomy of *Xt *mice. Homozygotes die by birth and the most prominent defects are found in the distal limbs, in rostral portions of the head including the forebrain and eyes (see below), along the ventral midline of the thorax and along the midline of the visceral ectoderm. Heterozygotes are described as developing relatively normally in all respects bar the formation of an extra digit, or digit-like appendage, on either the fore- or hindlimbs.

The vertebrate transcription factors Gli1, Gl2 and Gli3 are homologues of the *Drosophila *transcription factor cubitus interruptus [5]. These transcription factors transduce the responses of cells to diffusible morphogens of the Hedgehog family in both vertebrates and invertebrates [6]. Although Gli3 may have an early activator function in vertebrates [7], it is thought that its main actions are to repress the expression of sonic hedgehog (Shh) target genes. Thus, loss of Gli3 in mice that also lack Shh can reverse many of the defects associated with reduced hedgehog signalling [8], presumably because loss of Gli3 allows the reactivation of Shh target genes.

With regards the requirement for Gli3 in eye development,*Gli3*^-/- ^embryos exhibit a variety of eye abnormalities ranging from microphthalmia to the absence of any remnant of eye tissue [4,7,9-11]. Most likely these defects stem from interference with normal Shh signalling, which is essential for normal initiation of eye development [12] and later neural retinal development [13]. The extent to which a normal gene dosage of *Gli3 *is needed for eye development is not clear. The only eye defect described in mice heterozygous for mutations in *Gli3 *is the presence of folded retinae in a proportion of neonatal animals [4]. In the present study, we characterized the eye phenotype of *Gli3*^+/- ^mice and identified previously undescribed defects that were subtle. We then considered whether the requirement for a normal dosage of *Gli3 *might be greater if the gene dosage for another transcription factor known to be critical for eye development was to be reduced.

Extensive work on Pax6 has highlighted the important role that this molecule plays in both invertebrate and vertebrate eye development [e.g. reviewed in [14,15]]. Mutations within the *Pax6 *gene cause eye abnormalities in both mouse and man [16,17]. Mice homozygous for null mutations in the *Pax6 *gene fail to form eyes [16,18,19] whereas mice heterozygous for *Pax6 *mutations exhibit microphthalmia and have hypoplastic irises and multiple anterior segment defects [19-22]. There is evidence that Pax6 is both regulated by and regulates expression of Shh in brain tissue: Shh has been shown to repress Pax6 expression [23] and loss of Pax6 in mutant mice causes an upregulation of *Shh *expression [24-26]. Thus, reduction of either Pax6 or Gli3 levels might be predicted to affect eye development by activation, through different mechanisms, of Shh signalling. When we compared *Pax6*^+/-^; *Gli3*^+/- ^eyes to the eyes of wild-type, *Pax6*^+/- ^or *Gli3*^+/- ^siblings we observed a range of abnormalities more extensive than in *Gli3*^+/- ^or *Pax6*^+/- ^mutants or than would be predicted by addition of their phenotypes.

## Results

### Overlapping expression of Pax6 and Gli3 in the developing eye

**Figure 1:**
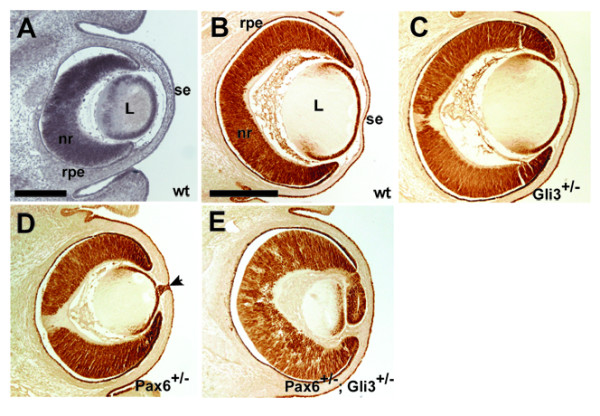
(A) *Gli3 *mRNA expression in the developing eye at E14.5. mRNA expression is observed in the neural retinal, retinal pigment epithelium, lens and surface ectoderm. (B-E) Pax6 protein expression in (B) wild type, (C)*Gli3*^+/-^, (D) *Pax6*^+/- ^and (E) *Pax6*^+/-^; *Gli3*^+/- ^eyes. In wild type embyos (B), Pax6 is expressed in cells of the neural retina, lens epithelium and surface ectoderm. *Gli3*^+/-^embryos (C) show identical Pax6 expression to wild type with no overt retinal dysmorphology. Eyes of the developing *Pax6*^+/- ^embryo (D) show a similar Pax6 expression to wild type but cells forming the persistent lens stalk (arrow) can be clearly seen to be Pax6 expressing. *Pax6*^+/-^;*Gli3*^+/- ^eyes (E) exhibit Pax6 expression localised to the neural retina, retinal pigment epithelium and lens epithelium but show overt distal retinal dysplasia. Rpe, retinal pigment epithelium; nr, neural retina; L, lens; se, surface ectoderm. Scale bar = 200 μm.

As shown in Fig. 1A,B, Pax6 and Gli3 are expressed in the prenatal mouse eye [see also [5,18,27,28]]. Both genes are expressed in the neural retina, retinal pigment epithelium, lens and surface ectoderm.

### General development of the eye in *Gli3*^+/-^, *Pax6*^+/- ^and *Gli3*^+/-^; *Pax6*^+/- ^mutants

The appearances of E14.5 *Gli3*^+/-^, *Pax6*^+/- ^and *Gli3*^+/-^;*Pax6*^+/- ^eyes, immunostained for Pax6 expression, are shown in Fig. 1C–E. Pax6 immunoreactivity was seen in the developing neural retina, retinal pigment epithelium, lens and surface ectoderm in all geneotypes, as in wild-types. There were, however, differences in morphology.

The E14.5 Gli3^+/- ^eyes appeared slightly larger than those of wild-type siblings, although their appearances were otherwise normal (Fig. 1C). The E14.5 *Pax6*^+/- ^eyes appeared smaller than those of wild-type siblings (Fig. 1D) and Pax6 protein was seen in cells forming a lens-corneal bridge (arrowhead in Fig. 1D), as described previously [22]. The sizes of E14.5 *Pax6*^+/-^; *Gli3*^+/- ^eyes were more variable than those of siblings of the other three genotypes; many were similar to those of *Pax6*^+/- ^eyes (Fig. 1E). *Pax6*^+/-^; *Gli3*^+/- ^eyes showed more obvious abnormalities than eyes of the other genotypes, including dysplasia at the most distal tips of the retina causing ectopic retinal tissue within the presumptive anterior chamber (Fig. 1E).

**Figure 2:**
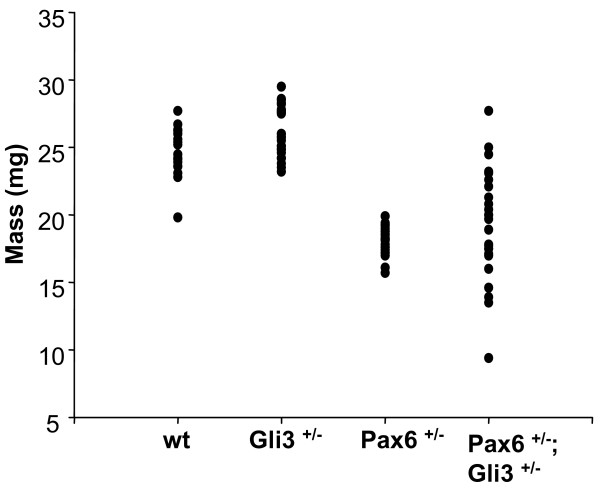
Masses of wild-type (n = 18), *Gli3*^+/- ^(n = 30), *Pax6*^+/- ^(n = 24) and *Pax6*^+/-^; *Gli3*^+/- ^(n = 25) adult eyes.

We then examined the overall mass of eyes of the different genotypes in adults (Fig. 2). Similar to previous studies [22], we found that the average mass of *Pax6*^+/- ^eyes (18.1 ± 0.2 mg, *sd *= 1.0, *σ*^2 ^= 1.0, n = 24) was significantly less than that of wild-type eyes (24.7 ± 0.4 mg, *sd *= 1.8, *σ*^2 ^= 3.4, n = 18) (*P *< 0.001, Student's t-test) (Fig. 2). The average mass of *Gli3*^+/- ^eyes was slightly, but significantly, larger than that of wild-type eyes (26.1 ± 0.3 mg, *sd *= 1.8, *σ*^2 ^= 3.1, n = 30; *P *= 0.016, Student's t-test) (Fig. 2). The average mass of *Pax6*^+/-^; *Gli3*^+/-^eyes (19.3 ± 0.8 mg, *sd *= 4.1, *σ*^2 ^= 16.7, n = 25) was significantly less than that of wild-type eyes (*P *< 0.001, Student's t-test), but not significantly different to that of *Pax6*^+/- ^eyes. There was much greater variation than among eyes of the other genotypes (Fig. 2). Whereas only 1/24 *Pax6*^+/- ^eyes fell within the range for wild-type eyes, 12/25 of *Pax6*^+/-^; *Gli3*^+/- ^eyes fell within the wild-type range and 4/25 eyes weighed less than the smallest mass observed in the *Pax6*^+/- ^group (Fig. 2).

### *Pax6*^+/-^; *Gli3*^+/- ^animals exhibit severe retinal abnormalities

**Figure 3:**
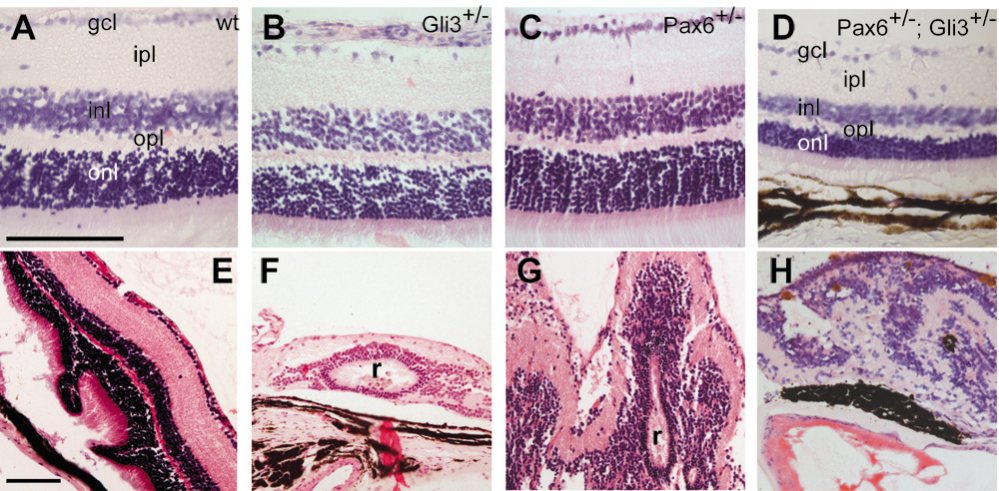
Retinal morphology of wild-type (A), *Gli3*^+/- ^(B, E), *Pax6*^+/- ^(C) and *Pax6*^+/-^;*Gli3*^+/- ^(D, F-H) animals. Although a majority of *Gli3*^+/- ^retinae exhibit normal lamination (B), mild focal dysplasia is seen (E). *Pax6*^+/-^; *Gli3*^+/- ^retinal phenotypes include thinning of the retina (D) and dysplasia (F-H). gcl, ganglion cell layer; inl, inner nuclear layer; onl, outer nuclear layer; ipl, inner plexiform layer; opl, outer plexiform layer; r, rosette. Scale bars = 100 μm.

No retinae from wild-type littermates of our mutant animals showed any overt abnormalities (Fig. 3A). The majority of *Gli3*^+/- ^animals (22/25) showed no retinal dysplasia (Fig. 3B); a small proportion (3/25) did exhibit focal retinal hyperplasia (Fig. 3E). No retinal dysplasia was observed in any of the *Pax6*^+/- ^eyes studied (Fig. 3C) (n = 14). *Pax6*^+/-^; *Gli3*^+/- ^retinae fell into 2 categories. 1) Some retinae exhibited no dysplasia but were thinner than those of other genotypes (15/28) (Fig. 3D). 2) Some retinae were dysplastic (13/28) (Fig. 3F–H) with areas where lamination was completely disrupted with loss of any defined retinal organisation and mixing of the nuclear layers.

**Figure 4:**
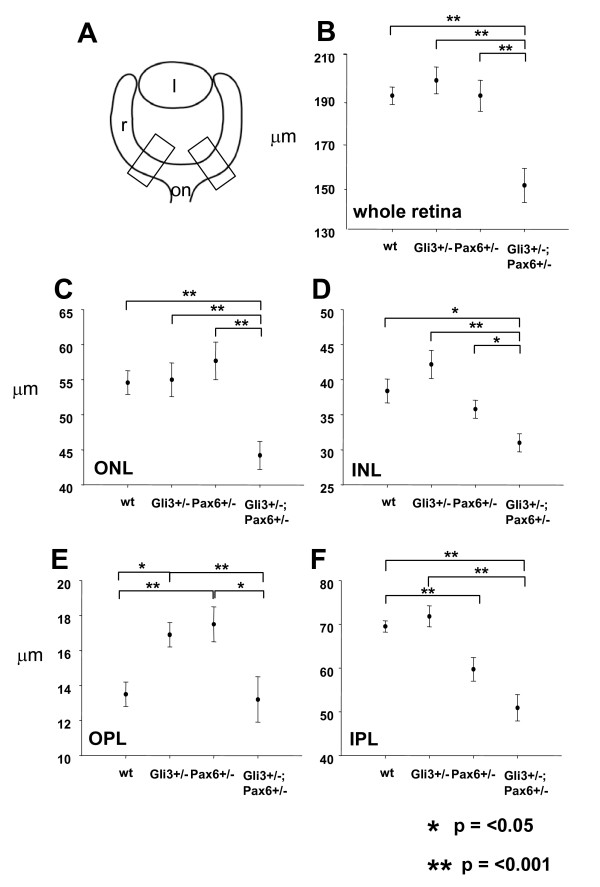
Retinal layer thickness in wild-type, *Gli3*^+/-^, *Pax6*^+/- ^and *Pax6*^+/-^; *Gli3*^+/- ^eyes. (A) Schematic representation of areas of retinal layer measurements in wild-type and mutant eyes. Measurement of retinal layers for (B) whole retinal thickness, (C) outer nuclear layer (ONL), (D) inner nuclear layer (INL), (E) outer plexiform layer (OPL), (F) inner plexiform layer (IPL) showing average thickness (μm) (+/- s.e.m.) for each genotype group. Significant differences between genotypes are shown with brackets. l, lens; r, retina; on, optic nerve. * *P *= < 0.05; ** *P *= 0.001.

We compared retinal thicknesses in the positions shown in Fig. 4A in wild-type, *Pax6*^+/-^, *Gli3*^+/- ^and *Pax6*^+/-^;*Gli3*^+/- ^animals. Compound heterozygotes that had retinal dysplasia in the regions measured were excluded. Whole retinal thickness was significantly reduced in *Pax6*^+/-^;*Gli3*^+/- ^animals compared to their wild-type, *Pax6*^+/- ^and *Gli3*^+/- ^siblings, all of which had similar thicknesses (Fig. 4B).

We then examined which retinal layers were most affected in *Pax6*^+/-^; *Gli3*^+/- ^retinae. The adult neural retina is divided into 5 layers: the outer nuclear layer (ONL), the outer plexiform layer (OPL), the inner nuclear layer (INL), the inner plexiform layer (IPL) and the ganglion cell layer (GCL). We found that the thicknesses of both the ONL and INL of *Pax6*^+/-^; *Gli3*^+/- ^retinae were significantly reduced compared to those of the other genotypes (Fig. 4C,D). In some *Pax6*^+/-^; *Gli3*^+/- ^retinae (3/21), the OPL was not discernible; where this layer was present, its average thickness was not significantly different to that of wild-type animals (Fig. 4E). Interestingly, the OPLs of both *Pax6*^+/- ^and *Gli3*^+/- ^retinae were thicker than those of wild-type and *Pax6*^+/-^; *Gli3*^+/- ^animals (Fig. 4E). The IPL of the *Pax6*^+/-^; *Gli3*^+/- ^retina was significantly thinner than that of wild-type and *Gli3*^+/- ^retinae, but was not significantly different to that of *Pax6*^+/- ^animals (Fig. 4F), which was itself significantly thinner than that of wild-types (Fig. 4F). As the GCL is only one cell thick, measurement was not done on this layer. We did not, however, observe any obvious defects in the GCL in any of the genotype groups. Overall, we concluded that the thicknesses of the retina and most of its layers are severely compromised in *Pax6*^+/-^; *Gli3*^+/- ^animals but not in *Pax6*^+/- ^or *Gli3*^+/- ^siblings.

### Anterior segment and lens defects in *Pax6*^+/-^; *Gli3*^+/- ^animals

**Figure 5:**
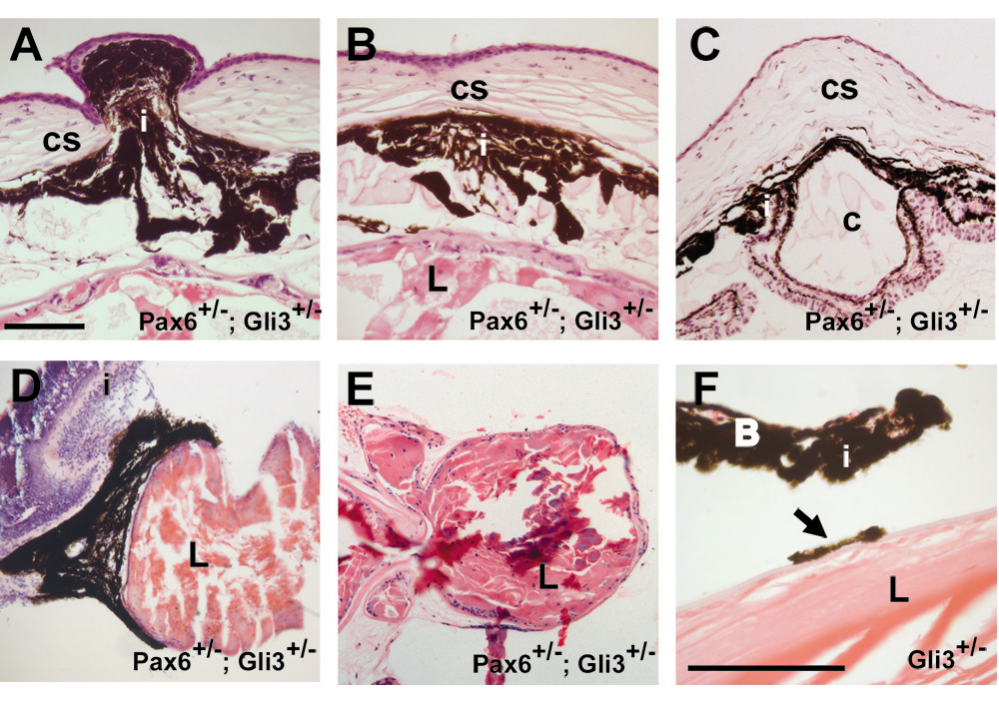
Anterior segment abnormalities in *Pax6*^+/-^; *Gli3*^+/- ^eyes. Abnormal contacts between cornea and iris (A-C), lens and cornea (E) and iris and lens (D) were observed in *Pax6*^+/-^; *Gli3*^+/- ^eyes. (A-C) Iris hyperplasia and formation of cyst-like structures involving iris, retina and cornea were also observed (C). (D, E) Some lenses of *Pax6*^+/-^; *Gli3*^+/- ^animals were highly dysgenic. Some *Gli3*^+/- ^animals had iris tissue attached to the lens (arrow in F). i, iris; cs, corneal stroma; L, lens; c, cyst. Scale bars = 100 μm.

Consistent with previous studies [22], we observed lens-corneal adhesions with cellular bridges in *Pax6*^+/- ^embryonic (Fig. 1D) and adult eyes (Table 1). Lens-corneal adhesions were found with a similar incidence in *Pax6*^+/-^; *Gli3*^+/- ^eyes (Table 1). We observed several other types of abnormal contact in *Pax6*^+/-^; *Gli3*^+/- ^eyes, which were not found in wild-type, *Pax6*^+/- ^or *Gli3*^+/- ^eyes in our sample. These were common between the cornea and iris (Fig. 5A,B) but were also seen between retina and lens and iris and retina (Table 1). Some *Gli3*^+/- ^eyes showed small points of contact between the iris and lens (Fig. 5F; Table 1); these were not seen in *Pax6*^+/-^eyes and were more frequent and extensive in *Pax6*^+/-^; *Gli3*^+/- ^eyes (Table 1). We can not be sure of the exact nature of these contacts, but the fact that we did not observe them in wild-type eyes suggests that they reflect real defects in the interactions between different parts of the eye.

**Table 1:** Incidence of abnormal contacts between different tissues in the eyes of wild-type, *Gli3*^+/-^, *Pax6*^+/- ^and *Pax6*^+/-^; *Gli3*^+/- ^animals.

**Genotype**	**Eyes n =**	**Cornea-lens**	**Cornea-iris**	**Iris-lens**	**Retina-lens**	**Iris-retina**
**Wt**	17	0%	0%	0%	0%	0%
***Gli3*^**+/-**^**	25	0%	0%	**28%**	0%	0%
***Pax6*^**+/-**^**	16	**38%**	0%	0%	0%	0%
***Pax6*^**+/-**^; *Gli3*^**+/-**^**	28	**36%**	**64%**	**68%**	**39%**	**7%**

Many *Pax6*^+/-^; *Gli3*^+/- ^eyes exhibited highly dysgenic hyperplastic irises (12/28) (Fig. 5A,B), which were not observed in any of the other genotype groups. Large cyst-like structures were observed between the iris, retina and overlying cornea (Fig. 5C) in one *Pax6*^+/-^; *Gli3*^+/- ^eye. Some *Pax6*^+/-^; *Gli3*^+/- ^animals (9/28) showed highly dysmorphic and dysgenic lenses, which were not observed in any of the other genotype groups (Fig. 5D, E).

### Corneal defects in *Pax6*^+/-^; *Gli3*^+/- ^animals

**Figure 6:**
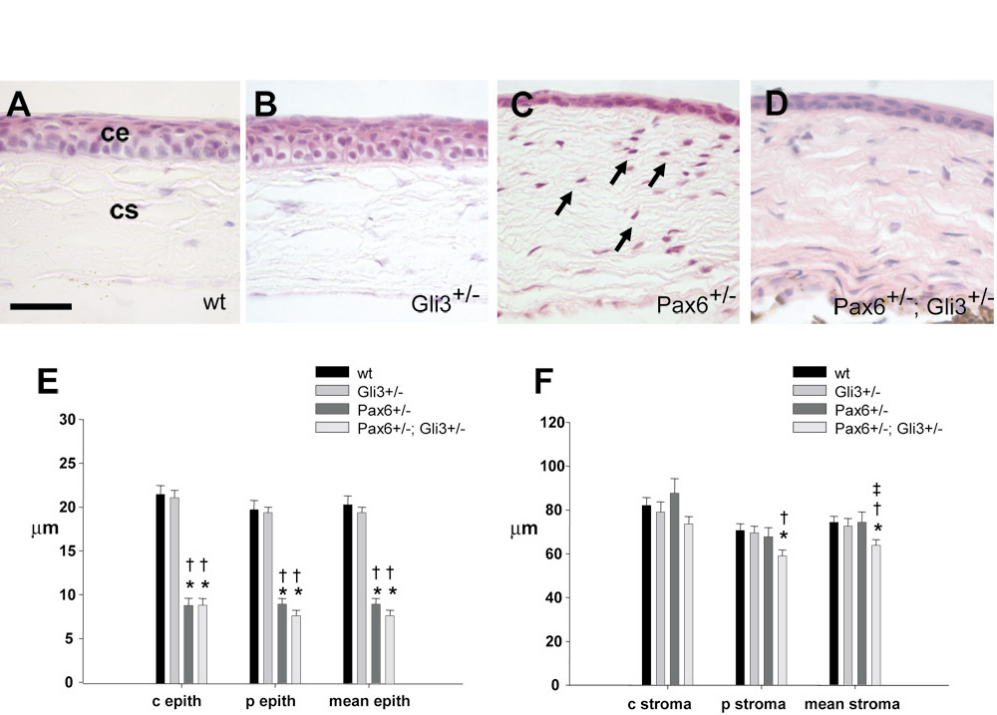
Corneal abnormalities in *Pax6*^+/- ^and *Pax6*^+/-^; *Gli3*^+/- ^eyes. Corneal morphology of (A) wild-type, (B) *Gli3*^+/-^, (C) *Pax6*^+/- ^and (D) *Pax6*^+/-^; *Gli3*^+/- ^animals. (C) *Pax6*^+/- ^and (D) *Pax6*^+/-^; *Gli3*^+/- ^eyes have a thinner corneal epithelium and more hypercellular stroma (arrows in C) than wild-type (A) and *Gli3*^+/- ^(B) eyes. Graphs show relative thicknesses of (E) corneal epithelium and (F) corneal stroma of the various genotype groups. The corneal epithelium of *Pax6*^+/- ^and *Pax6*^+/-^; *Gli3*^+/- ^eyes was significantly thinner than that of wild-type and *Gli3*^+/-^eyes, both centrally and peripherally. The peripheral stroma of *Pax6*^+/-^; *Gli3*^+/- ^eyes was thinner than that of wild-type and *Gli3*^+/- ^eyes, thus making the mean stromal thickness (average of peripheral and central) of *Pax6*^+/-^; *Gli3*^+/- ^eyes significantly less than that of wild-type, *Pax6*^+/- ^and *Gli3*^+/- ^eyes. * = significantly different to wild-type; † = significantly different to *Gli3*^+/-^; ‡ = significantly different to *Pax6*^+/-^. ce, corneal epithelium; cs, corneal stroma. Scale bar = 50 μm.

The corneal epithelium of the *Pax6*^+/- ^eye has been reported to be thinner than normal and the corneal stroma is hypercellular [21,22]. We confirmed these findings in our samples (Fig. 6A,C). At the gross level, no differences were observed in the cornea between wild-type and *Gli3*^+/- ^animals (Fig. 6A,B). *Pax6*^+/-^; *Gli3*^+/- ^animals exhibited similar corneal abnormalities to those observed in *Pax6*^+/- ^animals (Fig. 6C,D).

Thicknesses of the corneal epithelium and corneal stroma, centrally and peripherally, were measured for the four genotype groups. There was no difference in both central or peripheral corneal epithelium thickness between wild-type and *Gli3*^+/- ^eyes (Fig. 5E). As previously described [22], the corneal epithelium of *Pax6*^+/- ^eyes (n = 19) was significantly thinner than that of wild-type (n = 16) and *Gli3*^+/- ^(n = 27) eyes, both centrally and peripherally (Fig. 6E; compared to wild-type and *Gli3*^+/-^: both *P *< 0.001, Student's t-test). A similar result was observed in *Pax6*^+/-^; *Gli3*^+/- ^eyes (n = 25) (Fig. 6E; compared to wild-type and *Gli3*^+/-^: both *P *< 0.001; Student's t-test). Overall, these data suggest that loss of one copy of both *Pax6 *and *Gli3 *together does not create a corneal epithelium thinner than that resulting from loss of *Pax6 *alone.

There was no difference in either central or peripheral corneal stromal thickness in *Pax6*^+/- ^or *Gli3*^+/- ^eyes compared to wild-type eyes (Fig. 6F). The peripheral stroma of *Pax6*^+/-^; *Gli3*^+/- ^eyes was, however, slightly thinner than that of wild-type and *Gli3*^+/- ^eyes, while the central stroma was not different to that of wild-type eyes (Fig. 6F; compared to wild-type: *P *= 0.009; compared to *Gli3*^+/-^: *P *= 0.016; Student's t-tests). Thus, the mean stromal thickness (average of peripheral and central) of *Pax6*^+/-^; *Gli3*^+/- ^eyes was significantly less than that of wild-type (Fig. 6F; *P *= 0.010, Student's t-test), *Pax6*^+/- ^(Fig. 6F; *P *= 0.042, Student's t-test) and *Gli3*^+/- ^(Fig. 6F; *P *= 0.049, Student's t-test) eyes.

## Discussion

The importance of the transcription factors *Pax6 *[16,18,19] and *Gli3*[4,9,10] in eye development is reflected in the severe eye phenotypes of homozygous mutants, which die around the time of birth. While it is well documented that loss of one copy of *Pax6 *results in various eye defects [20-22], little is known about whether loss of one copy of *Gli3 *results in eye defects. Thus, in this study, we set out to characterise the morphology of the *Gli3*^+/- ^eye. Furthermore, because both *Pax6 *and *Gli3 *are expressed in the optic cup/retina, lens and iris during development [5,18,27-29], we were also interested in whether *Pax6 *and *Gli3 *play mutually co-operative roles in eye formation by studying animals compound heterozygous for mutations in both *Pax6 *and *Gli3*.

No abnormality was observed in the majority of the *Gli3*^+/- ^eyes that we examined. Where abnormalities were seen, they were very mild. *Gli3*^+/- ^eyes had a slightly higher mass than wild-type eyes as well as a thicker OPL compared to wild-type retinae. Retinal folding has been described previously in some neonatal *Gli3*^+/- ^animals [4]. Overall, defects in *Gli3*^+/- ^eyes are either not detectable or minor.

Animals compound heterozygous for mutations in both *Pax6 *and *Gli3 *exhibited unique and more severe eye defects than either *Pax6*^+/- ^or *Gli3*^+/- ^animals or than would be predicted by addition of their phenotypes. As previously described [22], the mass of *Pax6*^+/- ^adult eyes was significantly smaller than that of wild-type eyes. The additional loss of one copy of *Gli3 *resulted in eyes with a wide range of eye mass, from some eyes with smaller mass than *Pax6*^+/- ^eyes to some eyes which fell within the wild-type range of eye mass.

**Figure 7:**
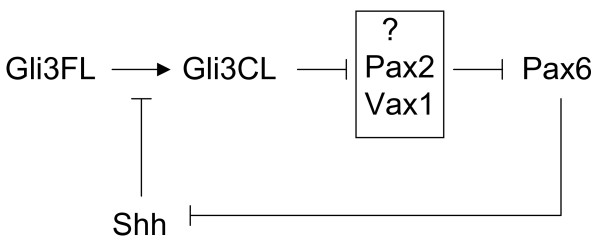
Summary of possible interactions between Gli3 and Pax6 in Shh signalling. The processing of Gli3 to its cleaved form (Gli3CL) from its full-length form (Gli3FL) is inhibited by Shh. Levels of Pax6 are regulated by levels of Gli3CL by a process of double repression, perhaps involving Pax2 and Vax1, such that lowering levels of Gli3CL lowers Pax6 levels. Pax6 negatively regulates Shh expression: thus, lowered Pax6 levels increase Shh's repression of Gli3CL production.

A range of retinal phenotypes was observed in *Pax6*^+/-^; *Gli3*^+/- ^eyes. Some *Pax6*^+/-^; *Gli3*^+/- ^retinae were thinner than normal but exhibited normal lamination while others were severely dysgenic with considerable laminar dysmorphology. The severe retinal defects found in *Pax6*^+/-^; *Gli3*^+/- ^animals were never seen in either *Pax6*^+/- ^or *Gli3*^+/- ^animals. One mechanism by which *Pax6 *and *Gli3 *might cooperate during retinal development is through their actions in the Shh signalling pathway. This pathway is critical for normal eye development. Shh from the ventral midline of the early neural plate stage embryo is required for early patterning of the visual system [12]. Later, Shh is expressed in the developing retina [30] and has been shown to be important in photoreceptor development and retinal lamination [13,31]. Gli3 is essential for the transduction of Shh signalling: in the absence of Shh, the Gli3 protein is cleaved proteolytically to produce a form that is thought to function mainly as a repressor [32]. Lowering the gene dosage of *Gli3 *can partially rescue the Shh-null phenotype [8], suggesting that reducing Gli3 levels allows the partial reactivation of Shh signalling pathways. It is possible, therefore, that a reduced level of Gli3 in otherwise wild-type cells (i.e. in *Gli3*^+/- ^mice) mimics the effects of raising Shh levels. There is evidence that Shh suppresses Pax6 expression [23,33], and lowered Pax6 expression is known to affect eye development. While the loss of one copy of *Gli3 *(mimicking raised Shh signalling) might not lower Pax6 levels enough to create morphologically detectable defects if both alleles of *Pax6 *are functional, levels might fall below a critical threshold in *Pax6*^+/-^;*Gli3*^+/- ^heterozygotes. Furthermore, there is evidence that Pax6 suppresses Shh expression [24-26] and so the decreased levels of Pax6 in the *Pax6*^+/-^;*Gli3*^+/- ^heterozygotes might allow increased production of Shh, thereby further exacerbating the overstimulation of the Shh pathway. The mechanism by which reduced levels of the repressor form of Gli3 result in reduced Pax6 levels might involve loss of repression of intermediate transcription factors such as Pax2 and Vax1, that themselves repress Pax6 [11]. These possibilities are summarized in Fig. 7. While an interaction in Shh signalling seems likely, there are numerous other signalling pathways in which Gli3 and Pax6 might cooperate. A strong possibility is in fibroblast growth factor 8 (FGF8) signalling, since both Gli3 and Pax6 are known to regulate FGF8 expression [29,34]

Like the defects in the retina, abnormalities in the anterior segment of the *Pax6*^+/-^; *Gli3*^+/- ^animals were more severe than those of either *Pax6*^+/- ^or *Gli3*^+/- ^animals. *Pax6*^+/-^; *Gli3*^+/- ^animals had highly dysgenic lenses which were never seen in *Pax6*^+/- ^or *Gli3*^+/- ^animals. There were particularly high incidences of abnormal contacts involving the iris in *Pax6*^+/-^; *Gli3*^+/- ^animals and, in contrast to the iris hypoplasia observed in *Pax6*^+/- ^animals, many *Pax6*^+/-^; *Gli3*^+/- ^animals exhibited hyperplastic irises. Together these data suggest not only that the iris is sensitive to levels of *Pax6 *expression but that normal *Gli3 *expression may also be required, in conjunction with *Pax6*, for normal iris development to occur. *Pax6*^+/-^; *Gli3*^+/- ^animals also showed thinning of the corneal epithelium and disorganisation of the corneal stroma, similar to that observed in *Pax6*^+/- ^animals. Unlike *Pax6*^+/- ^and *Gli3*^+/- ^animals, however, the peripheral corneal stroma was significantly thinner than in wild-types. As *Gli3*^+/- ^animals showed no difference in the thickness of the corneal stroma, the reduction of *Gli3 *dosage alone does not affect stromal thickness but does so only in combination with reduced *Pax6 *dosage.

## Conclusion

We have found that animals compound heterozygous for mutations in both *Pax6 *and *Gli3 *have eye abnormalities that are either not present in, or are more severe than, those present in animals heterozygous for mutations in either gene. Addition of the phenotypes of *Pax6*^+/- ^and *Gli3*^+/- ^animals is insufficient to explain the severe phenotypes of the compound mutants. Our findings suggest that *Gli3 *and *Pax6 *cooperate during eye morphogenesis.

## Methods

### Mice

The *Pax6*^+/- ^(*Small eye*, *Sey*) strain was maintained on a mixed (C57Bl/Fa, Ju/Fa and JBT/Jd) background as described previously [35]. The *Gli3*^+/- ^strain (*Extra toes, Xt*^*J*^) was maintained on an inbred CBA/Ca background. Animals wild-type at the *Pax6 *and *Gli3 *loci, heterozygous for either mutation (*Pax6*^+/- ^or *Gli3*^+/-^) and compound heterozygous for mutations in both genes (*Pax6*^+/-^*; Gli3*^+/-^) were obtained by crosses between *Pax6*^+/- ^and *Gli3*^+/- ^animals. 65 animals from 17 litters were analyzed. All comparisons were between wild type, *Pax6*^+/-^*, Gli3*^+/- ^and *Pax6*^+/-^*; Gli3*^+/- ^siblings. Animals were killed by cervical dislocation; eyes were removed and some were weighed before fixing for histological preparation. All animal procedures were performed in accordance with Home Office (UK) legislation.

### Histology

Eyes were fixed in either 4% paraformaldehyde or Bouin's fluid overnight before dehydrating and processing to paraffin wax. Bouin's fluid was selected for tissue for morphometric analysis as it causes few artefacts of tissue fixation. Sections were cut at 10 μm and stained with haematoxylin and eosin. Midsections through the eye containing the optic nerve were identified for morphometric retinal and corneal analysis.

### Genotyping

Mutations in the *Pax6 *gene were assessed as described previously [35]. Mapping of the mutant *Gli3*^*XtJ *^allele has revealed a 51.5-kb deletion [36]. Using a multiplex PCR strategy [35], wild-type *Gli3 *and mutant *Gli3*^*XtJ *^alleles were identified using the following primers: 580For 5' TACCCCAGCAGGAGACTCAGATTAG-3' and 580Rev 5'-AAACCCGTGGCTCAGGACAAG-3'; C3For 5'-GGCCCAAACATCTACCAACACATAG-3' and C3Rev 5'-GTTGGCTGCTGCATGAAGACTGAC-3' producing products of 193 bp and 580 bp for the wild-type and mutant alleles respectively.

### Morphometric analysis

Morphometric analysis of the retinal layers was performed only in eye sections where normal laminar morphology of the retina was maintained. Animals with eyes which were severely dysplastic were not included in this analysis. Transverse eye sections at the level of the optic nerve (medial section) were photographed with a digital camera at ×40. The thicknesses of the whole retina as well as the thicknesses of the inner nuclear layer (INL), outer nuclear layer (ONL), inner plexiform layer (IPL) and outer plexiform layer (OPL) were measured using ImageTool™. Two separate measurements were taken on either side of the optic nerve (Fig. 4A) and averaged. Statistical comparison of layer thicknesses between genotype groups was carried out using Sigmastat™. Student's t-tests were used to compare layer thicknesses between genotype groups.

Analysis of corneal epithelial and stromal thickness was performed essentially as described previously [22]. Serial transverse sections of each eye were taken. The medial section ('Section 0') of each eye was determined. Analysis of corneal thickness was performed using a linear eye piece graticule on three medial and near-medial sections (Sections 0, -3 and +3). The thickness (apical-basal depth) of the corneal epithelium, stroma and the whole cornea were measured separately at the centre and periphery of the cornea on all three sections for each eye. Data from central and peripheral regions were combined to obtain overall mean thickness of corneal epithelium and stroma in each eye. Statistical comparison of corneal thickness between genotype groups was carried out using Sigmastat™. Student's t-tests were used to compare corneal layer thickness between genotype groups.

### Immunohistochemistry

Slides were microwaved in 10 mM sodium citrate to achieve maximal antigen retrieval before addition of primary antibody. Mouse monoclonal anti-Pax6 antibody (1:100) was obtained from the Developmental Studies Hybridoma Bank (University of Iowa, Department of Biological Sciences, Iowa City, IA 52242). Signal was enhanced using the Dako ABC Kit and visualised with diaminobenzidine (DAB).

### RNA in-situ hybridisation

A 611 base pair fragment comprising nucleotides 560–1170 of the mouse Gli3 cDNA (a gift from T. Theil) was PCR amplified and subcloned into a pGEM-Teasy vector (Promega). The plasmid was linearized with SpeI and transcribed with T7 RNA polymerase. Non-radioactive RNA in-situ hybridisation on paraffin wax-embedded sections was carried out using a protocol described previously [27].

## Abbreviations

GCPS Greig cephalopolysyndactyly

DAB diaminobenzidine

FGF8 fibroblast growth factor 8

GCL ganglion cell layer

INL inner nuclear layer

IPL inner plexiform layer

ONL outer nuclear layer

OPL outer plexiform layer

PHS Pallister-Hall Syndrome

Shh Sonic hedgehog

Xt extra-toes

## Authors' contributions

PZ and JQ designed and carried out most of the experiments, MC participated in the quantitative analysis, JT carried out in situ hybridizations, IS participated in the design and carrying out of genotyping, DP participated in the analysis and writing of the manuscript.

## References

[B1] Vortkamp A, Gessler M, Grzeschik KH (1991). GLI3 zinc-finger gene interrupted by translocations in Greig syndrome families. Nature.

[B2] Kang S, Graham JM, Olney AH, Biesecker LG (1997). GLI3 frameshift mutations cause autosomal dominant Pallister-Hall syndrome. Nat Genet.

[B3] Vortkamp A, Franz T, Gessler M, Grzeschik KH (1992). Deletion of Gli3 supports the homology of the human Greig Cephalopolysyndactyly Syndrome (GCPS) and the mouse mutant extra toes (Xt). Mammalian Genome.

[B4] Johnson DR (1967). Extra-toes: a new mutant gene causing multiple abnormalities in the mouse. J Embryol Exp Morphol.

[B5] Hui CC, Slusarski D, Platt KA, Holmgren R, Joyner AL (1994). Expression of three mouse homologs of the Drosophila segment polarity gene cubitus interruptus, Gli, Gli-2, and Gli-3, in ectoderm- and mesoderm-derived tissues suggests multiple roles during postimplantation development. Dev Biol.

[B6] Zaki PA, Martynoga B, Price DJ, Howie S, Fisher C (2005). The role of hedgehog and Gli signalling in telencephalic development. Shh and Gli signalling and development.

[B7] Tyurina OV, Guner B, Popova E, Feng J, Schier AF, Kohtz JD, Karlstrom RO (2005). Zebrafish Gli3 acts as both an activator and repressor in Hedgehog signalling. Dev Biol.

[B8] Litingtung Y, Chiang C (2000). Specification of ventral neuron types is mediated by an antagonistic interaction between Shh and Gli3. Nature Neuroscience.

[B9] Franz T, Besecke A (1991). The development of the eye in homozygotes of the mouse mutant Extra-toes. Anat Embryol (Berl).

[B10] Kondoh H, Janet Rossant (2002). Development of the Eye. Mouse development: Patterning, Morphogenesis and Organogenesis.

[B11] Furimsky M, Wallace VA (2006). Complementary Gli activity mediates early patterning of the mouse visual system. Developmental Dynamics.

[B12] Chiang C, Litingtung Y, Lee E, Young KE, Corden JL, Westphal H, Beachy PA (1996). Cyclopia and defective axial patterning in mice lacking Sonic hedgehog gene function. Nature.

[B13] Wang YP, Dakubo G, Howley P, Campsall KD, Mazarolle CJ, Shiga SA, Lewis PM, McMahon AP, Wallace VA (2002). Development of normal retinal organization depends on Sonic hedgehog signaling from ganglion cells. Nat Neurosci.

[B14] Wawersik S, Maas RL (2002). Vertebrate eye development as modeled in Drosophila. Hum Mol Genet.

[B15] Ashery-Padan R, Gruss P (2001). Pax6 lights-up the way for eye development. Curr Opin Cell Biol.

[B16] Hill RE, Favor J, Hogan BLM, Ton CCT, Saunders GF, Hanson IM, Prosser J, Jordan T, Hastie ND, Vanheyningen V (1991). Mouse small eye results from mutations in a paired-like homeobox- containing gene. Nature.

[B17] Prosser J, van Heyningen V (1998). PAX6 mutations reviewed. Hum Mutat.

[B18] Grindley JC, Davidson DR, Hill RE (1995). The role of Pax-6 in eye and nasal development. Development.

[B19] Hogan BL, Horsburg G, Cohen J, Hetherington CM, Fisher G, Lyon MF (1986). Small eyes (Sey): a homozygous lethal mutation on chromosome 2 which affects the differentiation of both lens and nasal placodes in the mouse. J Embryol Exp Morphol.

[B20] Baulmann DC, Ohlmann A, Flugel-Koch C, Goswami S, Cvekl A, Tamm ER (2002). Pax6 heterozygous eyes show defects in chamber angle differentiation that are associated with a wide spectrum of other anterior eye segment abnormalities. Mech Dev.

[B21] Davis J, Duncan MK, Robison WG, Piatigorsky J (2003). Requirement for Pax6 in corneal morphogenesis: a role in adhesion. J Cell Sci.

[B22] Ramaesh T, Collinson JM, Ramaesh K, Kaufman MH, West JD, Dhillon B (2003). Corneal abnormalities in Pax6+/- small eye mice mimic human aniridia-related keratopathy. Invest Ophthalmol Vis Sci.

[B23] Macdonald R, Barth KA, Xu Q, Holder N, Mikkola I, Wilson SW (1995). Midline signalling is required for Pax gene regulation and patterning of the eyes. Development.

[B24] Stoykova A, Treichel D, Hallonet M, Gruss P (2000). Pax6 modulates the dorsoventral patterning of the mammalian telencephalon. J Neurosci.

[B25] Grindley JC, Hargett LK, Hill RE, Ross A, Hogan BL (1997). Disruption of PAX6 function in mice homozygous for the Pax6Sey-1Neu mutation produces abnormalities in the early development and regionalization of the diencephalon. Mech Dev.

[B26] Pratt T, Vitalis T, Warren N, Edgar JM, Mason JO, Price DJ (2000). A role for Pax6 in the normal development of dorsal thalamus and its cortical connections. Development.

[B27] Collinson JM, Quinn JC, Hill RE, West JD (2003). The roles of Pax6 in the cornea, retina, and olfactory epithelium of the developing mouse embryo. Dev Biol.

[B28] Walther C, Gruss P (1991). Pax-6, a murine paired box gene, is expressed in the developing CNS. Development.

[B29] Aoto K, Nishimura T, Eto K, Motoyama J (2002). Mouse GLI3 regulates Fgf8 expression and apoptosis in the developing neural tube, face, and limb bud. Dev Biol.

[B30] Jensen AM, Wallace VA (1997). Expression of Sonic hedgehog and its putative role as a precursor cell mitogen in the developing mouse retina. Development.

[B31] Black GC, Mazerolle CJ, Wang Y, Campsall KD, Petrin D, Leonard BC, Damji KF, Evans DG, McLeod D, Wallace VA (2003). Abnormalities of the vitreoretinal interface caused by dysregulated Hedgehog signaling during retinal development. Hum Mol Genet.

[B32] Ruiz i Altaba A (1999). Gli proteins encode context-dependent positive and negative functions: implications for development and disease. Development.

[B33] Li H, Tierney C, Wen L, Wu JY, Rao Y (1997). A single morphogenetic field gives rise to two retina primordial under the influence of the prechordal plate. Development.

[B34] Reza HM, Yasuda K (2004). The involvement of neural retina pax6 in lens fiber differentiation. Dev Neurosci.

[B35] Quinn JC, West JD, Hill RE (1996). Multiple functions for Pax6 in mouse eye and nasal development. Genes Dev.

[B36] Maynard TM, Jain MD, Balmer CW, LaMantia AS (2002). High-resolution mapping of the Gli3 mutation extra-toes reveals a 51.5-kb deletion. Mamm Genome.

